# Supplementary figures and images for: Dishevelled Has Anti-Viral Activity in Rift Valley Fever Virus Infected Aedes aegypti
Source: Viruses. 2023 Oct 24;15(11):2140. doi: 10.3390/v15112140 (PMC10675198; doi:10.3390/v15112140)

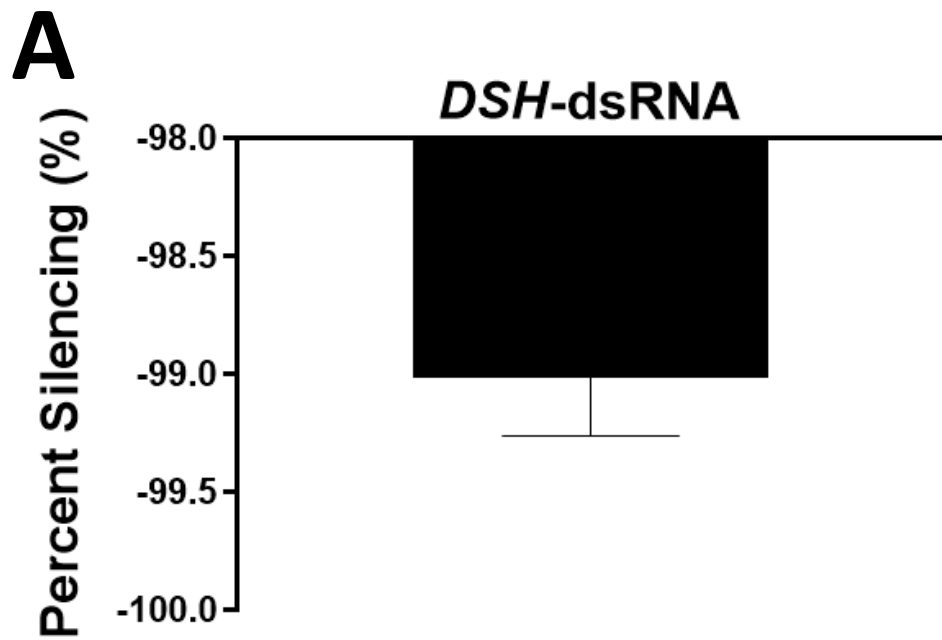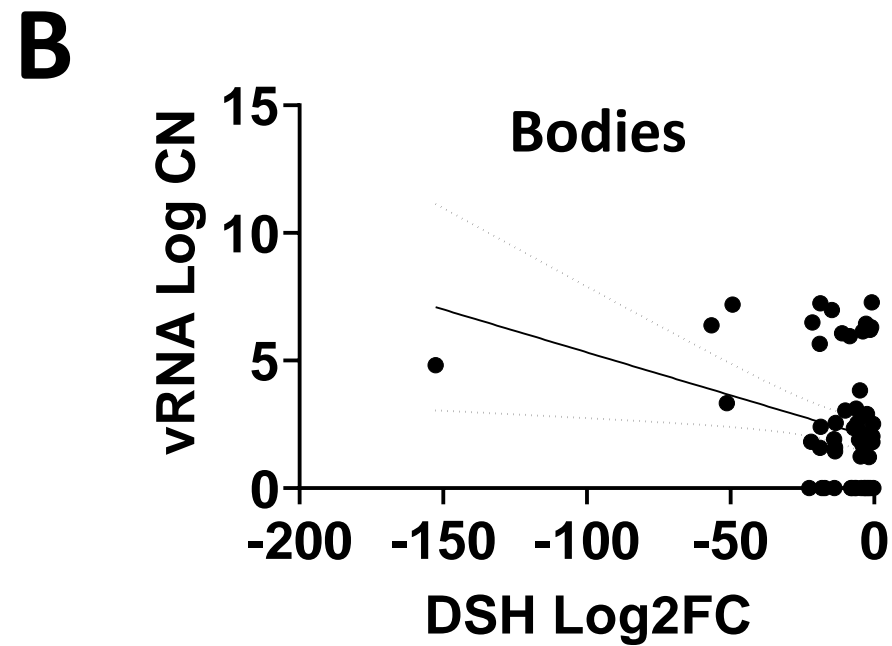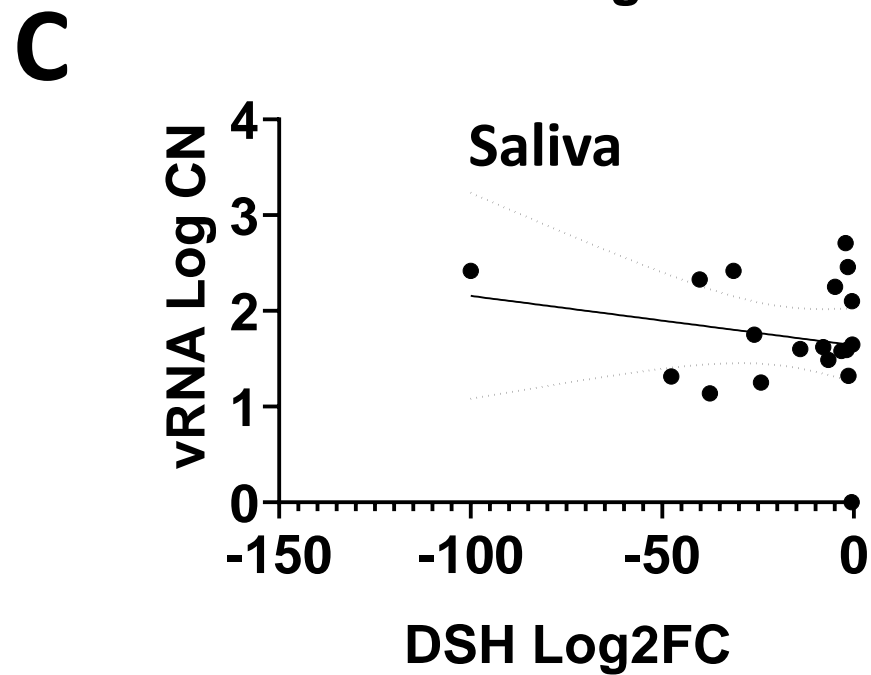

Supplement: Supplementary file 1 [file viruses-15-02140-s001.zip › Suppl Fig 1.pdf]
